# Left Ovarian Cyst With Overlying Sigmoid Colon

**DOI:** 10.7759/cureus.28927

**Published:** 2022-09-08

**Authors:** Henry Zou, Maggi Forgue

**Affiliations:** 1 Obstetrics and Gynecology, Michigan State University College of Human Medicine, Grand Rapids, USA; 2 Obstetrics and Gynecology, Trinity Health, Grand Rapids, USA

**Keywords:** ovarian cancer screening, ovarian cancer, sigmoid colon, laparoscopic treatment, ovarian torsion, ovarian cyst

## Abstract

Ovarian cysts are usually asymptomatic and self-resolvable, but large cysts can increase the risk of ovarian torsion or rupture and may be indicated for surgical intervention. We present the case of a large left ovarian cyst in which laparoscopic left salpingo-oophorectomy was challenged by an overlying sigmoid colon with dense adhesions. A 47-year-old female patient presented to the emergency department with abdominal pain in the right lower quadrant. Ultrasound and computed tomography scans found a large left ovarian cyst and multiple small right ovarian cysts. Due to the size of the left ovarian cyst increasing the risk for torsion, the patient was indicated for laparoscopic left salpingo-oophorectomy. However, the cyst was inaccessible due to the overlying sigmoid colon and dense adhesions on all sides. The surgeons elected to drain the cyst, and the patient was counseled that it was safe to monitor for postoperative recurrence over the next three months. Though laparoscopic surgery is considered a gold standard modality for minimally-invasive ovarian cystectomy/oophorectomy, our case illustrates how it can be challenging when treating left-sided adnexal masses in post-hysterectomy patients due to rectosigmoid and adhesional obstruction. In the context of this challenge, our case further demonstrates the importance of preoperative ovarian cancer screening and favoring conservative treatment options whenever possible.

## Introduction

Ovarian cysts describe fluid-filled sacs in the ovary that can develop during the menstrual cycle, are usually asymptomatic, and often self-resolve [1]. 18% of postmenopausal women in the US experience an incidence of ovarian cyst over 15 years, and 7% of women globally experience an incidence of ovarian cyst during their lifetimes [1]. The American College of Obstetricians and Gynecologists (ACOG) deems simple cysts safe to monitor via repeat ultrasound (US) [1]. However, some ovarian cysts can be malignant, and large cysts greater than 5 cm can increase the risk of ovarian torsion or rupture [2]. In these cases, surgical intervention may be indicated to avoid complications [1,2]. We present the case of a large left ovarian cyst in which laparoscopic salpingo-oophorectomy was challenged by an overlying sigmoid colon.

## Case presentation

**Figure 1:**
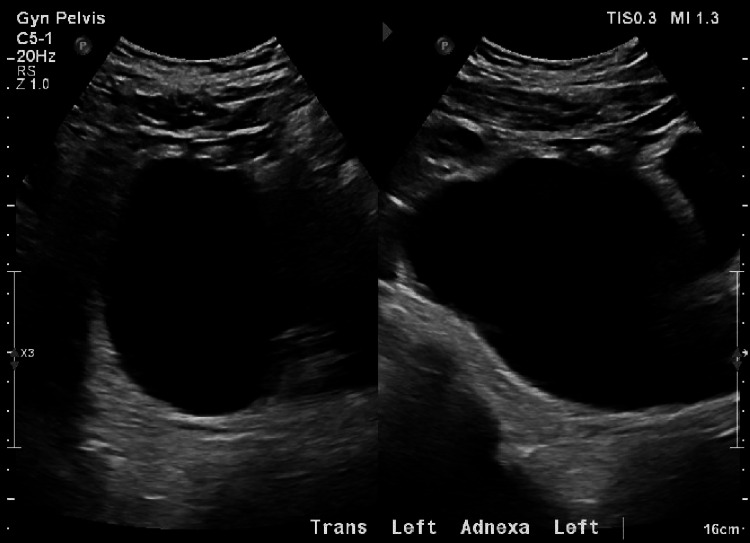
Left Ovarian Cyst on Transvaginal Pelvic Ultrasound

**Figure 2:**
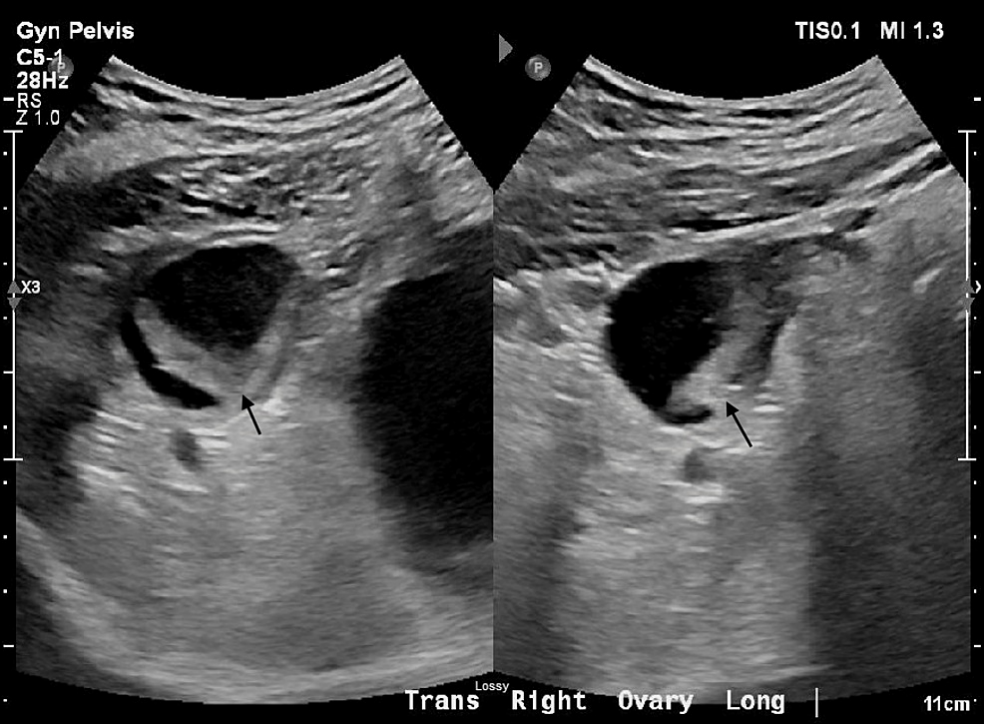
Right Ovarian Heterogeneity on Transvaginal Ultrasound Arrows indicate areas of heterogeneity

A 47-year-old female patient (G5P4014) with a history of hysterectomy and bilateral salpingectomy presented to the Emergency Department (ED) for a four-day history of sharp abdominal pain and decreased appetite. On physical exam, her abdominal pain was localized to the right lower quadrant (RLQ), which was also found to have tenderness, rebounding, and guarding. Transabdominal and the transvaginal US found a large left ovarian cystic mass (Figure 1) and right ovarian heterogeneity (Figure 2), but no ovarian torsion gave signs of decreased perfusion, thickened fallopian tubes, or twisted vascular pedicles/whirlpool signs. Abdominal and pelvic computed tomography (CT) scans with contrast further determined that the left ovarian cyst measured up to 12.4 cm in diameter, while the right ovary contained multiple small cysts that measured up to 2.5 cm in diameter. Given adequate visualization of the cysts in both ovaries, no further imaging was ordered. She was prescribed naproxen 500 mg twice daily for 15 days, but after six days of pain refractory to pharmacotherapy, she was admitted for hospitalization and referred for gynecologic evaluation.

She was seen by Obstetrics/Gynecology (OB/GYN) on the day of admission, where she was counseled that the large left ovarian cyst entailed a high risk of ovarian torsion and was indicated for a laparoscopic left salpingo-oophorectomy (LSO). Following a discussion of risks, the patient consented to the surgery and underwent Cancer Antigen 125 (CA125) and OVA1Ⓡ tumor biomarker testing for ovarian cancer; both results were within normal limits and indicated a low risk of malignancy.

LSO was scheduled and performed 20 days later; however, therapeutic laparoscopy found that the sigmoid colon lay overtop the suspected left ovarian mass (no photos of the surgery were taken). Attempts to take the sigmoid colon off the cyst revealed the presence of dense adhesions on all sides that made the left ovary inaccessible. General surgery was consulted, and attempts were made to remove adhesions on the left side wall. However, the adhesions were too dense, and no progress was made. Instead, the surgeons drained the left ovarian cyst using the harmonic scalpel under direct visualization, removing over 400 ccs of clear fluid; the drainage area was hemostatic. The surgeons deferred switching to open abdominal surgery or sending the drained fluid for cytology, given low concerns for ovarian cancer from previous tumor biomarker tests. After closing a 1 cm serosal injury to the sigmoid, achieving hemostasis, and closing the incisions, the patient was taken to the post-anesthesia care unit (PACU) in stable condition. 

The patient was healing well and denied complications at her two-week post-operative visit, and was counseled that re-attempting an LSO would likely also require a sigmoid resection and temporary ostomy. Given the low concern for ovarian cancer, the current plan is to monitor for recurrence of the cyst after drainage over the next three months via repeat pelvic US; CA125 and OVA1Ⓡ will also be repeated. If concerns for recurrence arise, then the patient will be referred to Gynecological Oncology to undergo evaluation for open or robot-assisted abdominal surgery.

## Discussion

Though ACOG considers most ovarian cysts benign, self-resolving, and safe to monitor using repeat ultrasonography and follow-up visits, cysts may be indicated for surgical intervention if they are large (>5 cm), causing pain, or suspicious for malignancy [3]. Furthermore, a history of laparoscopic hysterectomy is considered a risk factor for ovarian torsion [4]. Given our patient’s abdominal pain, large cyst size, and history of laparoscopic hysterectomy, we considered her at elevated risk for ovarian torsion and thus a viable candidate for surgical intervention via LSO. Minimally-invasive laparoscopic or robot-assisted surgery is currently considered the gold standard, with nearly 90% of ovarian cystectomies and oophorectomies being performed for benign indications using minimally-invasive methods in the US [5]. Laparoscopic surgery has lower rates of postoperative pain, complications, urinary tract infections, and febrile morbidity compared to laparotomy [6]. However, laparoscopic surgery can be particularly difficult for cases involving left-sided adnexal masses with a history of hysterectomy, as there can be obstruction from the rectosigmoid colon and its mesentery that cannot be resolved without resection [1]. Moreover, the feasibility of the operation can often only be determined after exploratory laparoscopy has been performed [1]. In rare cases, laparoscopic surgery can result in the spillage of malignant cells if a cancerous adnexal mass is ruptured; however, careful preoperative ovarian cancer screening procedures and the appropriate selection of surgical patients can minimize this risk [7]. Our case illustrates how laparoscopic surgery can be challenged by obstructions from the sigmoid colon and multi-directional adhesions in a post-hysterectomy patient, as well as the importance of performing thorough preoperative ovarian cancer screening to minimize the risk of laparoscopy-induced malignant metastasis. It was assumed that the adhesions were due to the hysterectomy rather than endometriosis given the patient's clinical presentation and lack of chocolate cysts per US, CT, and laparoscopy findings. Given the low risk for ovarian cancer and the limitations set by the patient’s informed consent, we opted to drain the cyst and practice postoperative watchful waiting rather than attempt sigmoid resection.

## Conclusions

We present the case of a large 12.4 cm-diameter left ovarian cyst in a post-hysterectomy patient in which LSO was challenged by an overlying sigmoid colon and dense adhesions. Our case highlights how laparoscopic surgery can be difficult due to rectosigmoid and adhesional obstruction, particularly in the context of left-sided ovarian cystectomy/oophorectomy in a post-hysterectomy patient. Moreover, our case illustrates the importance of practicing thorough preoperative ovarian cancer screening and favoring conservative treatment measures whenever possible.

## References

[1] Farghaly SA (2014). Current diagnosis and management of ovarian cysts. Clin Exp Obstet Gynecol.

[2] Bottomley C, Bourne T (2009). Diagnosis and management of ovarian cyst accidents. Best Pract Res Clin Obstet Gynaecol.

[3] (2007). ACOG Practice Bulletin. Management of adnexal masses. Obstet Gynecol.

[4] Mashiach R, Canis M, Jardon K, Mage G, Pouly JL, Wattiez A (2004). Adnexal torsion after laparoscopic hysterectomy: Description of seven cases. J Am Assoc Gynecol Laparosc.

[5] Dioun S, Huang Y, Melamed A (2021). Trends in the use of minimally invasive adnexal surgery in the United States. Obstet Gynecol.

[6] Medeiros LR, Fachel JM, Garry R, Stein AT, Furness S (2005). Laparoscopy versus laparotomy for benign ovarian tumours. Cochrane Database Syst Rev.

[7] Hulka JF, Parker WH, Surrey MW, Phillips JM (1992). Management of ovarian masses. AAGL 1990 survey. J Reprod Med.

